#  Radioprotective Effect of Thymol Against Salivary Glands Dysfunction Induced by Ionizing Radiation in Rats

**Published:** 2016

**Authors:** Seyed Mohammad Abedi, Fateme Yarmand, Mina Motallebnejad, Maryam Seyedmajidi, Dariush Moslemi, Ali Bijani, Seyed Jalal Hosseinimehr

**Affiliations:** a *Department of Radiology, Faculty of Medicine, Mazandaran University of Medical Sciences, Sari, Iran. *; b *Department of Oral Medicine, Faculty of Dentistry, Babol University of Medical Science, Babol, Iran.*; c *Cellular and Biology Research Center, Babol University of Medical Science, Babol, Iran.*; d *Dental Materials Research Center, Faculty of Dentistry, Babol University of Medical Science, Babol, Iran.*; e *Department of Radiotherapy, Faculty of Medicine, Babol University of Medical Science, Babol, Iran.*; f *Department of Physiology and Cellular and Molecular Biology Research Center, Babol University of Medical Science, Babol, Iran. *; g *Department of Radiopharmacy, Faculty of Pharmacy, Mazandaran University of Medical Sciences, Sari, Iran.*

**Keywords:** Thymol, Radioprotective, Salivary gland dysfunction, Scintigraphy, Radiotherapy

## Abstract

The aim of this study was to investigate the radioprotective effect of thymol as a natural product against salivary glands dysfunction induced by ionizing radiation in rats. The rats were treated with thymol at dose of 50 mg/Kg before exposure to ionizing radiation at dose 15 Gy. Salivary gland function was evaluated with radioisotope scintigraphy and then salivary gland to background counts ratio was calculated. Ionizing radiation caused significant salivary glands dysfunction at the 3^th^ and the 70^th^ days with reduction in radioactivity uptake in salivary glands. Ratios of salivary gland to background radioactivities were 2.0 ± 0.05, 1.58 ± 0.62 and 1.99 ± 0.07 at 3^th^ days for control, radiation, and thymol plus radiation groups, respectively. Thymol significantly protected acute and chronic salivary gland dysfunction induced by ionizing radiation in the rats.This finding may have been a promising application of thymol for the protection of salivary glands dysfunction induced by ionizing irradiation in patients exposed to radiation in head and neck cancer therapy.

## Introduction

Radiotherapy of head and neck cancer can cause severe adverse effects on oral cavity such as xerostomia and salivary gland dysfunction which impair quality life of patients undergoing radiation therapy (1-3). Ionizing radiation produces reactive oxygen species (ROS) and other toxic substances, which interact with critical macromolecules such as DNA, and leads to serious cellular dysfunction and death (4, 5). However, ionizing radiation (IR) is focusing on tumor tissue for killing cancerous cells; it may also cause side effects on the normal organs (6). The salivary glands are organs that are unwantedly exposed to radiation in the head and neck region. However, salivary glands are considered to be radio-resistance due to matured and differentiated cells; exposure to high dose of ionizing radiation such as head and neck radiotherapy or radioiodine therapy affects their functions (7-9). IR causes major changes on salivary gland and leads to salivary glands dysfunctions (10, 11). The main complications related to salivary gland dysfunction are including xerostomia, difficulty in swallowing and speaking, taste changes and high dental caries risk, which affect the patient’s quality of life (12, 13). Also salivary gland dysfunction is the most common side effect of oral β-emitting ^131^I for the treatment of well-differentiated thyroid carcinoma (DTC). The severity of salivary gland dysfunction increases with increase dosage of ^131^I radioiodine administration. Fallahi *et al.* reported that consumption of vitamin E may be associated with a significant protective effect against radiation-induced dysfunction in salivary glands following single-dose ^131^I therapy in patients with differentiated thyroid cancer (14). It is important to protect salivary glands against radiation-toxicity and also improves quality life of patients after radiotherapy. Antioxidants can scavenge free radicals and toxic substances produced by IR and have a beneficial role in protection of cellular macromolecules against toxicity induced by IR (15). Thymol is a natural phenolic compound that presents in various plants, such as thyme (Lamiaceae) and Zataria (16-18). Thymol has several biological properties such as anti-inflammatory activity (19) and protective effects against toxicity induced by oxidative stress in liver and lymphocytes (20-22). Thymol acts as an antioxidant, free radical scavenging and anti-lipid peroxidation, which can protect cell against free radicals (20, 23). With respect to these protective mechanisms, it is possible thymol protects salivary glands dysfunction caused by IR. Salivary scintigraphy is a useful technique to evaluate objectively the salivary gland function of patients with head and neck irradiated tumors. Salivary gland scintigraphy with Na^99m^TcO_4_ is a well-established procedure for the evaluation of human salivary gland function (24, 25). There is a good correlation between the scintigraphic findings and the measured salivary flow rates in evaluation of salivary gland function (26).

The aim of this study was to evaluate the radioprotective effects of thymol on the radiation-induced damages on salivary glands in the irradiated rats. This protective effect was evaluated by salivary gland scintigraphy with using Na^99m^TcO_4_. 

## Material and methods

*Animal treatment*

Male Wistar rats between 8 and 10 weeks old were used (Pasteur Institute, Iran). They were kept in polycarbonate cages under an alternating 12 h light-dark cycle in animal house of university. This study was approved by research and education deputy of Heart Center Hospital, Mazandaran University of Medical Sciences, Sari, Iran (5394). Animals were maintained on laboratory chow and water *ad libitum*. The animals were randomly divided into three groups of seven animals as control, radiation and thymol plus radiation. Control group (C) was received three doses of sesame oil given intraperitoneally 48, 24 and 1h; radiation group (IR) was treated with three doses sesame oil given intraperitoneally 48, 24 and 1h before irradiation. Thymol plus radiation group was received thymol at single dose 50 mg/Kg given intraperitoneally 48, 24 and 1h before the gamma irradiation. 

*Irradiation*

Thirty minutes before irradiations, the animals were weighed and anesthetized by an intramuscular injection of ketamine chlorhydrate (0.1 mg/Kg) and xylazin (0.05 mg/Kg). Animals were placed on the table with a 3 mm lead to protect the body, so that only the head and neck regions were exposed. The animals were irradiated with a cobalt-60 gamma radiation source (Theratron 780, AECL, Ontario, Canada) in Babolsar radiotherapy hospital with a single exposure to 15 Gy of radiation. 

*Salivary glands function assessment*

Salivary glands function assessments were performed in Heart Central Hospital (Sari, Iran) Ten minutes after IV injection (via tail*´*s vain) of Na^99m^TcO_4_ (^99^Mo/^99m^Tc generator, Pars isotope, Tehran, Iran), the rats placed on the gamma camera table for imaging. Salivary gland scintigraphy was acquired by a digital gamma camera (dual head, Siemens e.cam, Germany) with a matrix of 256×256 pixels, and using a pinhole collimator with a zoom factor of 1.78. Imaging started 10 min after injection of Na^99m^TcO_4_ and was conducted up to a quantity of 300 kilocounts. Imaging process was taken about five min. The processing ESOFT software was used on gamma camera. The uptake of the salivary glands was determined by a region of interest (27) technique. All ROIs were defined manually for each single rat and left and right gland region. The background activity was placed in the supraclavicular area. For the evaluation of glandular function, the ratio of the accumulation in the gland ROI to the accumulation in the background ROI, termed gland-to-background ratio was used. Static salivary gland scintigraphy scans were performed in three times; first scintigraphy was performed 7 days before the radiation or sham irradiation. The second and third scintigraphies were performed at 3 and 70 days after radiation or sham irradiation. 

*Statistical analysis*

Experimental data were expressed as the mean ± standard deviation (SD). The results were comparedwith the control group, and statistical analysis was performed by independent t-test to determine the significance of the difference between groups.The differences were considered significant when the P value was*<*0.05.

## Results

**Figure 1:**
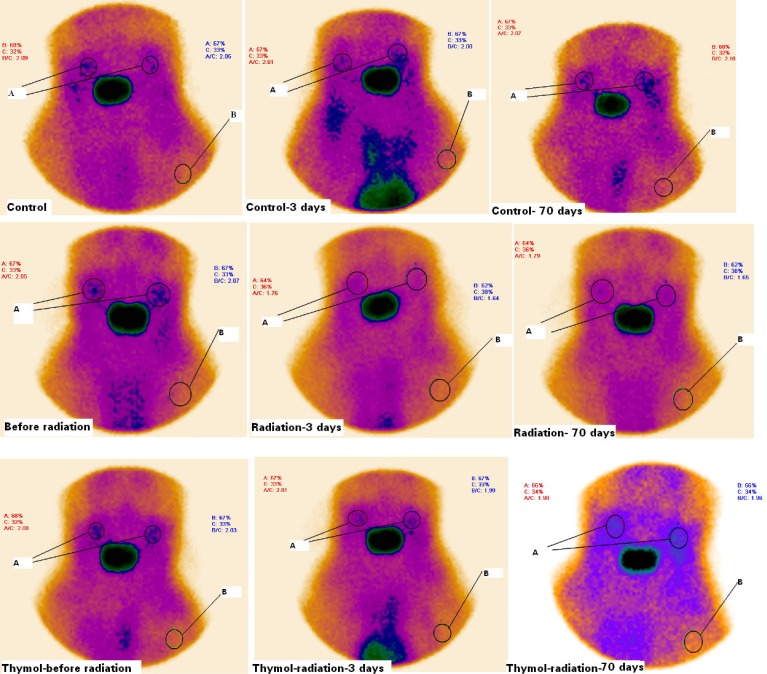
Scintigraphy of salivary glands of rats treated with thymol (50 mg/Kg) and/ or radiation on the before and 3th and 70th days after irradiation. Rats were treated with oil or thymol and then irradiated with gamma irradiation at dose of 15 Gy. A significant reduction in salivary glands uptake of radioactive was observed in irradiated rats as compared with control and thymol groups. A: salivary glands (T), B: background (B). T/B ratios were calculated with respect to radioactivity accumulation in salivary glands and background

Rats were exposed to gamma ray at a single dose 15 Gy on the oral region. All rats were alive up to end of the study. Static salivary gland radioisotope scintigraphy was performed for assessment of any tissue dysfunction caused by ionizing radiation in the rats. Scintigraphy scans of salivary glands are shown in Figure 1. Radioisotope uptake was quantified in salivary glands of rats. In this study, the target tissue was salivary glands and salivarygland-to-background ratios (T/B ratio) were determined in the treated groups with irradiation and/or thymol (Figure 1.). The T/B ratio is about two for rats before exposed to radiation. It was not observed any significant difference between the groups before the gamma radiation exposure in T/B ratios (Table 1). 

**Table 1:** Salivary gland-to-background ratios of treated groups in scintigraphy assessment (n=7).

	Before Radiation Mean ± SD	3 days after radiation Mean ± SD	70 days after radiation Mean ± SD
Control	2.04±0.07 *	2±0.5	1.99±0.11
Radiation	2.03±0.04 *	1.58±0.62 **	1.58±0.08**
Thymol + radiation	2.12±0.1*	1.99±0.07 ***	2.01±0.1***

* Non-significant differences between control, radiation and thymol+radiation groups before radiation

** Significant difference between control and radiation groups

*** Significant difference between thymol+radiation and radiation groups

Exposure to irradiation exhibited a significant salivary gland dysfunction that was shown a reducing in T/B ratio. IR reduced the uptake of radioisotope in salivary glands. The T/B ratios were 2 ± 0.5 and 1.58 ± 0.62 on the 3^th^ day and 1.99 ± 0.11and 1.58 ± 0.08 on the 70^th^ day for control and IR groups, respectively (Table 1). Thymol increased the T/B ratio up to 1.99 ± 0.07 and 2.01 ± 0. 4 in the irradiated rats treated with thymol compared to the gamma-irradiated rat alone on the 3^th^ day and the 70^th^ day. It is clear that thymol treatment increased uptake of Na^99m^TcO_4_in salivary glands and it improved salivary gland hypofunction caused by irradiation. 

## Discussion

In this study, the radioprotective effect of thymol against salivary glands dysfunction induced by ionizing radiation was investigated using radioisotope scintigraphy scans. The salivary gland to background radioactivity counts ratio was calculated. The right and left salivary glands, and background ROI were used for the calculation of the target-to-background ratio (26). In static scintigraphy, an increased uptake of radioisotope was seen in the salivary glands in the Wistar rats. Thymol at three doses of 50 mg/Kg significantly protected the salivary glands hypofunction caused by ionizing radiation in rats. Thymol elevated uptake Na^99m^TcO_4_in salivary glands that reduced by ionizing radiation. In this study, local mouth exposure to radiation caused an acute salivary glands dysfunction on the 3^th^ day after the irradiation and the chronic organ impairment was continued up to 70^th^ days without any improvement. Ionizing radiation caused a severe salivary glands damage, which was not improved at long time. Radiation induces apoptosis in salivary glands cells and causes salivary glands hypofunction (28, 29). Ionizing radiation caused a reduction of saliva secretion, salivary amylase activity, and superoxide dismutase (30). In this study, a high dose of 15 Gy of gamma rays was used to test the properties of thymol as a radioprotector. It was previously demonstrated that high dose of ionizing radiation about 15 Gy starts to induce salivary gland dysfunction (31).

Our experiments showed that thymol protected acute and chronic side effects of irradiation on the salivary glands, which evaluated at the 3^th^ and the 70^th^ days after irradiation. Rats have a similar distribution of the sodium/iodide symporter with human, which suggests the suitability of this animal for scintigraphic studies of the salivary glands (32). Thymol as a monoterpene phenol compound has free radical scavenging and antioxidant properties. It directly scavenges free radicals produced by oxidative stress process in the cells as well as restored antioxidant capacities of cells through increasing of cell glutathione (33). Thymol suppressed radiation-induced genotoxicity, apoptosis, and necrosis in the lung fibroblast cell. These protective effects are related to free radical scavenging and modulation of oxidative stress by thymol (34). Thymol pre-treatment enhanced reduced intercellular defense enzymes such as glutathione, glutathione-S-transferase, catalase, and superoxide dismutase levels in mouse liver homogenates after exposure to radiation. Thymol treatment before exposure to ionizing radiation resulted in a significant increase in hematological parameters (21). Thymol can protect genotoxicity induced by ionizing radiation on human lymphocyte cells (35). The phenolic structure of thymol is playing a crucial role in absorbing and neutralizing free radicals (23, 36).

In conclusion, ionizing radiation caused salivary glands dysfunction in rats through a reduction of radioactivity uptake in salivary glands. This side effect was sever and continued for a long time on the 70^th^ day after irradiation. Thymol at dose 50 mg/Kg significantly improved salivary gland dysfunction caused by ionizing radiation. Short and late side effects of radiation on the salivary glands were protected by thymol in rats. Several previous studies were reported radioprotective effects of thymol in various tissues in animal and as well as in vitro. Thymol is a promising radioprotective agent for patients who receive radiation in head and neck cancer therapy.

## Conflict of interest statement

The authors declared no potential conflict of interest with respect to the authorship, and/or publication of this study.
